# Validation of the Chinese version of Morningness-Eveningness Questionnaire for screening delayed sleep-wake phase disorder with bipolar disorder among Han Chinese population

**DOI:** 10.3389/fpsyt.2026.1779010

**Published:** 2026-04-30

**Authors:** Ting Yang, Yuchen Lin, Tong Li, Zhaoyu Gan

**Affiliations:** Department of Psychiatry (Mental Health), the Third Affiliated Hospital of Sun Yat-Sen University, Guangzhou, China

**Keywords:** affective disorder, bipolar disorder, circadian rhythm, delayed sleep-wake phase disorder, Morningness-Eveningness Questionnaire

## Abstract

**Objective:**

Delayed sleep-wake phase disorder (DSWPD) is commonly seen in bipolar disorder (BD). However, valid screening tools for DSWPD have not been available, especially in China. Therefore, this study aimed to explore the validation of the Morningness-Eveningness Questionnaire (MEQ) for screening DSWPD with BD among Han Chinese population.

**Methods:**

**A total of** 267 patients with bipolar disorder and 130 healthy controls were screened with the Chinese version of Morningness-Eveningness Questionnaire (MEQ) for DSWPD. Of these, 49 completed the re-test after 2 weeks. The DSWPD diagnosis was based on clinical interview with the patients and related informants by trained psychiatrists according to the diagnostic criteria of the International Classification of Sleep Disorders (3rd edition, ICSD-3). Cronbach’s α coefficient, split-half reliability coefficient, and the Intra-class Correlation Coefficient (ICC) were calculated to assess the reliability of MEQ. The receiver operating characteristic curve (ROC) was plotted to evaluate MEQ performance. The optimal cut-off was chosen by maximizing the Youden index.

**Results:**

The internal consistency, split-half, and retest reliabilities of the MEQ were 0.758, 0.719, and 0.661, respectively. Three factors were obtained for the MEQ scale based on Exploratory factor analysis, with a cumulative factor loading of 37.384%. BD patients with DSWPD had significantly lower MEQ scores (36.85 ± 6.40) compared to BD patients without DSWPD (47.44 ± 7.39) and healthy controls (47.96 ± 7.28) (both p<0.001). The area under the ROC curve (AUC) was 0.867. The Youden index was the highest when a MEQ score of 43 was used as the cut-off point, with a sensitivity of 0.83 and a specificity of 0.75.

**Conclusion:**

The Chinese version of MEQ is a valid screening tool for DSWPD among bipolar disorder patients.

## 1 Introduction

Bipolar disorder (BD) is a common, debilitating psychiatric disorder often accompanied by sleep disturbances, particularly delayed sleep-wake phase disorder (DSWPD). A cross-sectional study from Norway found that approximately 10% of adult BD patients (42/404) also had delayed sleep phases (this study defined “delayed sleep” as taking ≥1 hour to fall asleep and sleeping ≥10 hours/day) (1). A study in Japan assessing 104 BD patients found that 35 (32.4%) met the diagnostic criteria for circadian rhythm sleep-wake disorder (CRSWD), of which 27 had DSWPD (2).

Previous studies have indicated that, compared to BD patients without DSWPD, those with DSWPD tend to be younger, have an earlier onset of the disorder, higher BMI, more mood episodes, a higher rate of rapid cycling, stronger suicide intents, higher comorbidity risks, and are more likely to be prescribed mood stabilizers and antidepressants. They also exhibit more severe symptoms and poorer social functioning (3–9). Thus, timely and accurate identification of DSWPD comorbid with BD is crucial for precise treatment.

Current sleep rhythm assessments include continuous core body temperature (CBT min) monitoring, dim light melatonin onset (DLMO) measurements, sleep diaries, actigraphy, and the Morningness-Eveningness Questionnaire (MEQ) (10). The CBT min and DLMO are capable of evaluating sleep rhythms (11, 12) and identifying endogenous circadian rhythms. However, they require trained, experienced staff and strict backgrounds for testing (the patient must measure for at least 6 hours (13). Sleep diaries and actigraphy can track 7 to 14 days of sleep cycles during both working and non-working days, but is made difficult by patient adherence. The reasons above made MEQ the best tool for clinical sleep rhythm assessment due to its accessibility, simplicity, and cost-effectiveness. The Chinese version of the MEQ, translated by Bin Zhang et al. (14), has demonstrated good reliability and validity. However, since the original study was conducted on Cantonese-speaking adults in Hong Kong aged 30.4~48.7 years (39.7 ± 4.6 years), its applicability to Mandarin-speaking populations in mainland China remains uncertain. Furthermore, no study has been done to assess the validation of MEQ for screening DSWPD among BD patients. Therefore, this study aims to analyze the reliability and validity of the Chinese version of MEQ in a Mandarin-speaking Han Chinese population and evaluate its performance in screening DSWPD among BD patients.

## 2 Material and methods

### 2.1 Participants

The subjects of this study were 267 patients with bipolar disorder from the psychiatric department of the Third Affiliated Hospital of Sun Yat-sen University between May 2023 and January 2024. 130 healthy controls were recruited during the same period by convenience sampling. The inclusion criteria for the bipolar disorder group (BD group) were: meeting the diagnostic criteria for bipolar disorder according to the 5th edition of the Diagnostic and Statistical Manual of Mental Disorders (DSM-5) (15); being Han Chinese; being aged between 12 and 45 years, having at least 6 years of education and being able to understand and cooperate with clinical assessments. Exclusion criteria included: confirmed organic brain damage or other unstable physical illnesses; uncooperative; having received electroconvulsive therapy within the past month or experiencing sleep rhythm disruption due to external factors. All BD patients met the inclusion criterion of a score ≥17 on the 24-item Hamilton Depression Rating Scale (HAMD) at enrollment. The inclusion criteria for the healthy control group (HC group) were: aged between 12 and 45 years; Han Chinese; being physically and mentally healthy as determined by medical history inquiries, physical examinations, and psychiatric evaluations; having at least 6 years of education and is able to understand and cooperate with clinical assessments. The exclusion criteria were the same as those for the bipolar disorder group.

This study protocol was approved by the ethics committee of the Third Affiliated Hospital of Sun Yat-sen University, with ethics approval number: II2023-188-02. Written informed consent was achieved from all the participants, and their guardians for those aged under 18.

### 2.2 Measurement

#### 2.2.1 The diagnosis of DSWPD and BD

The diagnosis of Delayed Sleep-Wake Phase Disorder (DSWPD) was primarily based on the diagnostic criteria of the International Classification of Sleep Disorders (3rd edition, ICSD-3). However, during the actual implementation, since some patients were already hospitalized or receiving medication upon recruitment, it would have been unethical to discharge them or to delay treatment and monitor them for at least 7 days (ideally 14 days) with a sleep diary or actigraphy for study inclusion. Therefore, in this study, the DSWPD diagnosis was based on clinical interview with patients and their related informants by trained psychiatrists, indicating that the patient had experienced a delayed sleep phase for at least 7 days (preferably 14 days, including both work and non-work days).

The diagnosis of BD was established through the structural clinical interview by trained psychiatrists with the structured clinical interview (SCID) for DSM-5 for bipolar disorder (16).

#### 2.2.2 The assessment of sleep rhythm

The sleep rhythm was assessed with the Chinese version of MEQ offline or online (https://www.wenjuan.com/edit/survey/6522083f0d12fef7b1e8494d?scene=assess#assess). The original MEQ was translated into Chinese language using the translation-back-translation method.

### 2.3 Statistical methods

Data analysis was performed using SPSS version 26.0. Normality test and homogeneity of variance test were conducted for quantitative data. For normally distributed data, results were presented as mean ± standard deviation (x̄ ± s), and intergroup comparisons were made using the independent sample t-test. For data not following a normal distribution, results were presented as median (Q_25_, Q_75_), and intergroup comparisons were made using non-parametric tests. Categorical variables were described as n (%), and intergroup comparisons were made using the chi-square test with Bonferroni correction. All tests were two-tailed, with *P* < 0.05 indicating statistical significance. Cronbach’s α coefficient was calculated to assess the internal consistency reliability of the MEQ, while the Spearman-Brown formula was used to calculate the split-half reliability coefficient. The test-retest reliability was evaluated using the Intra-class Correlation Coefficient (ICC). Exploratory factor analysis and principal component analysis were used, with factors extracted through orthogonal rotation via the maximum variance method to evaluate the structural validity of the questionnaire. The receiver operating characteristic (ROC) curve was plotted to assess the screening performance of MEQ. Its accuracy was calculated in terms of sensitivity and specificity for each theoretically possible cut-off. The optimal cut-off was determined by maximizing the Youden index (=sensitivity + specificity-1).

## 3 Results

### 3.1 Demographic characteristics and habit of psychoactive substance use of the participants

In the first round of measurement, a total of 397 valid questionnaires were collected, including 267 bipolar disorder (BD) patients aged 12 to 43 years, with an average age of 18.94 ± 5.7 years. Of these, 213 were female (79.8%). The healthy control (HC) group consisted of 130 individuals aged 15 to 55 years, with an average age of 21.3 ± 5.3 years. In the second round of measurement, 49 valid questionnaires were collected, with participants aged 14 to 42 years and an average age of 20.6 ± 5.8 years. Of these, 19 were male (38.8%), 5 were BD patients (10.2%), and 44 were healthy controls (89.8%).

Demographic characteristics and caffeine consumption habits of the study participants are presented in **Table 1** and **2**. As shown in **Table 1**, Compared to the HC group, the BD group had a higher proportion of females (79.8% vs. 60.0%, χ2 = 17.470, *P* < 0.001), a younger average age (18.94 ± 5.7 vs. 21.3 ± 5.3, t = -7.317, *P* < 0.001), and a lower level of education (a smaller proportion with undergraduate/college education or above, 26.2% + 1.9% vs. 90.0% + 6.9%, χ2 = 199.609, *P* < 0.001). A higher percentage of BD patients reported not having a habit of consuming caffeinated beverages (62.5% vs. 15.4%) and a higher percentage reported frequent caffeine consumption (7.9% vs. 6.2%) (χ2 = 93.757, *P* < 0.001). There were no statistically significant differences between the DSWPD+ and DSWPD- groups in terms of gender, age, educational level, or caffeine consumption habits (*P* > 0.05, **Table 2**).

**Table 1:** Intergroup comparison of demographic characteristics and psychoactive substance use: BD vs. HC.

Variable		BD (n=267)	HC (n=130)	χ²/t	P value
Gender	Male	54 (20.2%)	52 (40.0%)	17.470	<0.001*
	Female	213 (79.8%)	78 (60.0%)		
Age		18.94 ± 5.7	21.3 ± 5.3	-7.317	<0.001*
Education	Junior high school	45(16.9%)	0(0.0%)	199.609	<0.001*
	High school/technical high school	147(55.1%)	4(3.1%.)		
	Undergraduate/college	70(26.2%)	117(90.0%)		
	Postgraduate and above	5(1.9%)	9(6.9%.)		
Habit of caffeine consumption	Never	167(62.5%)	20(15.4%)	93.757	<0.001*
	Seldom (<4 times/month)	71(26.6%)	92(70.8%)		
	Often (1–3 times/week)	21(7.9%)	8(6.2%)		
	Frequent (5–7 times/week)	7(2.6%)	6(4.6%)		
	Daily (>1 time/day)	1(0.4%)	4(3.1%)		

Data are presented as n (%) unless otherwise indicated. P values were calculated using independent t-test for age and chi-square test for categorical variables.

*indicates a statistically significant result (*P* values <0.05).

**Table 2:** Demographic characteristics and psychoactive substance use: BD-DSWPD+ vs. BD-DSWPD-.

Variable		BD-DSWPD+ (n=142)	BD-DSWPD- (n=125)	χ²/t	P value
Gender	Male	25 (17.6%)	29 (23.2%)	1.290	0.256
	Female	117 (82.4%)	96 (76.8%)		
Age		18.5 ± 4.6	19.5 ± 6.8	-0.253	0.800
Education	Junior high school	23 (18.4%)	22 (15.5%)	2.891	0.409
	High school/technical high school	72 (57.6%)	75 (52.8%)		
	Undergraduate/college	27 (21.6%)	43 (30.3%)		
	Postgraduate and above	3 (2.4%)	2 (1.4%)		
Habit of caffeine consumption	Never	84 (59.2%)	83 (66.4%)	5.826	0.213
	Seldom (<4 times/month)	38 (26.8%)	33 (26.4%)		
	Often (1–3 times/week)	13 (9.2%)	8 (6.4%)		
	Frequent (5–7 times/week)	6 (4.2%)	1 (0.8%)		
	Daily (>1 time/day)	1 (0.7%)	0 (0.0%)		

Data are presented as n (%) unless otherwise indicated. P values were calculated using independent t-test for age and chi-square test for categorical variables.

### 3.2 No statistically significant differences were observed between the two subgroups.Reliability of the scale

#### 3.2.1 Internal consistency and split-half reliability

The overall Cronbach’s α coefficient for the 19 items of the MEQ scale was 0.758. After sequentially deleting items 1 through 19, the remaining scale’s Cronbach’s α coefficients ranged from 0.729 (item 19) to 0.769 (item 12). The correlation coefficients between the individual item scores and the total scale score ranged from 0.014 (item 14) to 0.541 (item 18). The split-half reliability coefficient was 0.719.

#### 3.2.2 Test-retest reliability

Two weeks after the initial test, 49 randomly selected participants were retested. The average score for the first test was 49.12 ± 7.30, while the average score of the retest was 47.18 ± 9.8. The correlation coefficient between the two measurements was 0.661 (*P* < 0.001). Overall, the scale demonstrated good temporal stability and consistency.

### 3.3 Structural validity analysis of the scale

**Figure 1:**
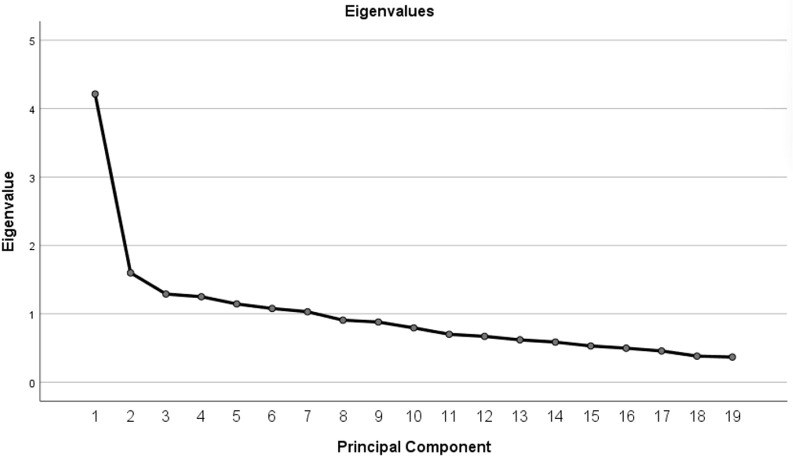
Scree plot.

The KMO value of the MEQ scale was 0.785, and the chi-square value for Bartlett’s test of sphericity was 1428.841 (*P* < 0.001), indicating that factor analysis was appropriate. As shown in the scree plot (**Figure 1**), the curve leveled off after the third factor, and the first three factors exhibited eigenvalues greater than 1. Based on the inflection point of the scree plot, the Kaiser criterion (eigenvalue >1), and the interpretability of the factors, a three-factor solution was retained, accounting for 37.384% of the cumulative variance. **Table 3** shows the factor loadings for each item. Except for items 8, 11, 14, 15, and 16, which had factor loadings less than 0.45, the factor loadings for all other items were greater than 0.45.

**Table 3:** Exploratory factor analysis of factor loadings for each item.

Items		Factor 1 (Wake Time)	Factor 2 (Sleep Time)	Factor 3 (Sleep Autonomy)
5	What is your mental state within half an hour of waking up in the morning?	0.739		
7	How do you feel within half an hour of waking up in the morning?	0.721		
4	How easy is it for you to wake up in the morning? (When you are not woken up by sudden events)	0.544		
17	Suppose you can choose your working hours, and you only need to work for 5 hours a day (including breaks). The job is interesting, and the salary is based on your performance. Which period would you choose?	0.535		
9	Suppose you decide to start exercising, and your friend suggests you should exercise for 1 hour twice a week, with 7-8am being the best time. Keeping in mind only your body clock, how do you think you would perform?	0.526		
19	People are often categorized as “morning types” or “night types.” Which type do you think you belong to?	0.504		
3	If you need to wake up at a certain time in the morning, how dependent are you on an alarm clock to wake you up?	0.494		
15	Suppose you have to do a strenuous physical task for 2 hours. You are completely free to plan your time and only need to consider your body clock. Which period would you choose?	0.422		
8	If you have no appointments the next day, compared to your usual sleeping schedule, what time would you choose to go to bed?	0.410		
14	Suppose you have to stay up late due to an urgent matter, and you need to stay awake from 4-6am. You have no appointments the next day. Which situation would suit you best?	0.233		
2	If you could freely plan your night, what time would you like to go to bed?		0.662	
1	If you could freely plan your daytime, what time would you like to wake up?		0.641	
13	Suppose for some reason you go to bed several hours later than usual, but you don’t need to wake up at a specific time the next morning. What is most likely going to happen?		0.495	
18	During which time of the day do you feel at your best?		0.467	
11	Suppose you want to perform your best on a mentally exhausting test that lasts for two hours. If you can freely plan your time and only need to consider your body clock, which period would you choose to take the test?		0.395	
16	Suppose you decide to start exercising, and your friend suggests that you should exercise twice a week for one hour, with the best time being between 10 - 11pm. Please remember that you only need to consider your biological clock. How do you think you will perform?		0.287	
10	At night, around what time do you start feeling tired and think you need to sleep?			0.677
12	If you went to bed at 11 pm, how tired would you be?			0.672
6	Do you feel hungry within half an hour of waking up?			0.642

### 3.4 Validity analysis of the scale

#### 3.4.1 Comparison of MEQ scores between groups

Overall, the MEQ score of the BD patient group was lower than that of the HC group (41.81 ± 8.67 vs. 47.96 ± 7.28, t=6.9787, *P* < 0.001). Specifically, the MEQ score of the BD-DSWPD+ group was lower than that of the HC group (36.85 ± 6.40 vs. 47.96 ± 7.28, *P* < 0.001), while there was no significant difference in MEQ score between the BD-DSWPD- group and the HC group (47.44 ± 7.39 vs. 47.96 ± 7.28, *P*>0.05). The MEQ score of the BD-DSWPD- group was higher than that of the BD-DSWPD+ group (47.44 ± 7.39 vs. 36.85 ± 6.40, *P* < 0.001*) (**Table 4**).

**Table 4:** Between-group comparisons of MEQ scores.

Variable	BD (n=267)	HC (n=130)	Statistical value/*P* ^1^	BD-DSWPD+(n=142)	BD-DSWPD- (n=125)	Statistical value/*P*^2^	Statistical value/*P^3^*	Statistical value/*P*^4^	Statistical value/*P^5^*
MEQ (x̄ ± s)	41.81 ± 8.67	47.96 ± 7.28	t=6.9787, *P* < 0.001*	36.85 ± 6.40	47.44 ± 7.39	F=109.345 *P* < 0.001*	10.588, *P* < 0.001*	-11.109, *P* < 0.001*	-0.522, *P* = 0.553

*indicates a statistically significant result (*P* values <0.05).

#### 3.4.2 ROC curve analysis of MEQ

**Figure 2:**
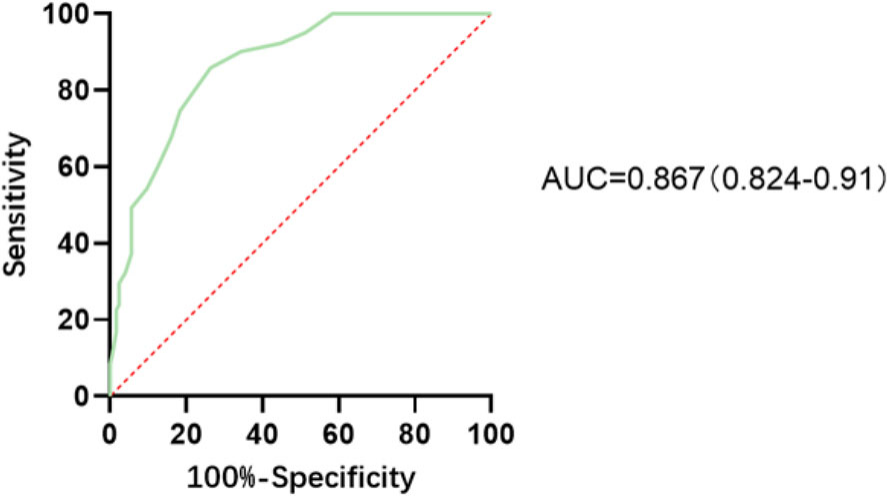
ROC curve. Note: AUC = 0.867 (95% CI: 0.824–0.910).

A logistic regression model was constructed using the total MEQ score to predict DSWPD status among BD patients. The AUC value was 0.867(95% CI: 0.824–0.910). When setting MEQ score of 43 as the cutoff point, the sensitivity was 0.859 (95% CI: 0.792–0.911), the specificity was 0.736(95% CI: 0.648–0.813), and the Youden index was the highest (0.595). (**Figure 2**; **Table 5**).

**Table 5:** Youden Index of MEQ scores.

Scores	Sensitivity	Specificity	Youden index
< 22.50	0.007042	1	0.007042
< 23.50	0.03521	1	0.03521
< 24.50	0.05634	1	0.05634
< 25.50	0.06338	1	0.06338
< 26.50	0.07042	1	0.07042
< 27.50	0.08451	1	0.08451
< 28.50	0.1197	0.992	0.1117
< 29.50	0.169	0.984	0.153
< 30.50	0.1901	0.984	0.1741
< 31.50	0.2254	0.984	0.2094
< 32.50	0.2394	0.976	0.2154
< 33.50	0.2958	0.976	0.2718
< 34.50	0.3239	0.96	0.2839
< 35.50	0.3732	0.944	0.3172
< 36.50	0.4225	0.944	0.3665
< 37.50	0.493	0.944	0.437
< 38.50	0.5423	0.904	0.4463
< 39.50	0.6056	0.872	0.4776
< 40.50	0.6761	0.84	0.5161
< 41.50	0.7465	0.816	0.5625
< 42.50	0.8028	0.776	0.5788
**< 43.50**	**0.8592**	**0.736**	**0.5952**
< 44.50	0.9014	0.656	0.5574
< 45.50	0.9225	0.552	0.4745
< 46.50	0.9507	0.488	0.4387
< 47.50	0.9718	0.456	0.4278
< 48.50	1	0.416	0.416
< 49.50	1	0.368	0.368
< 50.50	1	0.328	0.328
< 51.50	1	0.272	0.272
< 52.50	1	0.232	0.232
< 53.50	1	0.2	0.2
< 54.50	1	0.16	0.16
< 55.50	1	0.136	0.136
< 56.50	1	0.128	0.128
< 57.50	1	0.112	0.112
< 58.50	1	0.072	0.072
< 59.50	1	0.064	0.064
< 60.50	1	0.048	0.048
< 61.50	1	0.032	0.032
< 63.00	1	0.024	0.024
< 66.50	1	0.008	0.008

95% CI for sensitivity: 0.792–0.911; 95% CI for specificity: 0.648–0.813.

The bolded row (score < 43.50) indicates the optimal cut-off point determined by the maximum Youden index (0.5952).

## 4 Discussion

In this study, the item discrimination and homogeneity of the MEQ scale were satisfactory. The Cronbach’s α coefficient was 0.758, slightly higher than that reported in the study by Zhou et al. (17), suggesting that the MEQ has relatively high reliability among BD patients. Through exploratory factor analysis, three common factors were extracted, with a cumulative variance contribution rate of 37.384%. The number of factors is consistent with the Spanish version (10, 18) and the finding from the study on bipolar disorder by Keresa et al. (19), but slightly different from the Chinese version translated by Zhang Bin et al., which identified two factors (14). In addition, the factor contents also slightly differed from previous studies. In the Spanish version, there were three factors: Sleep Timing (items 1, 2, 10, 12, 14, and 16), Wake Timing (items 3, 4, 5, 7, 8, 9, and 13), and Most Efficient Time (items 6, 11, 15, 17, 18, and 19). In the Chinese version translated by Zhang Bin et al., the two factors were Sleep Phase and Best Performance Time. In this study, the three factors were Wake Time, Sleep Time, and Sleep Autonomy. Based on the item-factor loadings, most items showed good loadings, except for items 8, 11, 14, 15, and 16, which had poor loadings. The test-retest correlation coefficient of the MEQ after two weeks in this study was 0.661, similar to the results by Zhang Bin et al. (14), indicating that the scale is stable. Since DSWPD is a sleep disorder caused by a mismatch between endogenous circadian rhythms and the external environment, the three factors of Wake Time, Sleep Time, and Sleep Autonomy reflect the participant’s habitual/desired wake time, habitual/desired sleep time, and their willingness to determine their sleep rhythm based on external rhythmic demands (including social life, work, school, etc.). Therefore, we believe these three factors have certain significance in reflecting DSWPD and sleep rhythms. The cumulative variance explained by the three-factor solution in our study was 37.38%, which is lower than previous MEQ validation studies. This discrepancy may be attributable to differences in sample characteristics: our study included both BD patients and healthy controls, with the BD patients all in a depressive episode, whereas previous validation studies were conducted in general populations. Clinical samples typically exhibit greater heterogeneity, which naturally increases unexplained variance in factor analysis. Nevertheless, explained variance between 30-40% is often considered acceptable in exploratory factor analyses of psychological instruments in clinical populations, particularly when the factor structure has theoretical grounding. The three factors identified—Wake Time, Sleep Time, and Sleep Autonomy—are conceptually meaningful and directly relevant to the clinical features of DSWPD.

This study also found that BD patients with DSWPD had significantly lower MEQ scores compared to BD patients without DSWPD and healthy controls. Further, ROC curve analysis showed that using a MEQ score of 43 as the cutoff point could effectively identify DSWPD among BD patients, suggesting that the MEQ has good criterion validity for screening DSWPD. To our knowledge, this is the first study to evaluate the MEQ as a screening tool for DSWPD. In previous studies, MEQ scores below 41 were often used to distinguish whether an individual’s circadian rhythm belonged to the ‘evening type. A previous study even directly used MEQ scores below 41 as one of the diagnostic criteria for DSWPD. However, this approach lacks evidence, as ‘evening type’ and DSWPD are not equivalent (20), and their relationship requires further research.

Overall, this study found that the Chinese version of the Morningness-Eveningness Questionnaire (MEQ) has good reliability and validity, and can serve as an effective screening tool for identifying DSWPD among BD patients. However, several limitations should be considered when interpreting the findings of this study: 1) The BD patient group in this study was drawn from clinical patients, while the healthy control group was mainly recruited through advertisements, suggesting potential selection bias; 2) The diagnosis of DSWPD was not aided by sleep logs or actigraphy. Retrospective sleep assessments are susceptible to recall bias and subjective perception. The reliance on clinical interviews may partially explain the substantial variability in MEQ scores within the BD-DSWPD+ group. This group may have included subthreshold or atypical cases, thereby broadening the distribution of MEQ scores; 3) The included patients were currently undergoing medication treatment, which may have had some impact on their sleep rhythms. 4) The test-retest reliability analysis included only five patients with BD, which may limit the generalizability of the reliability estimate to the BD population. Future studies should consider using larger, more homogeneous samples to improve the scale’s structural validity in the Chinese BD population.

## Data Availability

The datasets generated and analyzed during the current study are not publicly available due to ethical restrictions and the protection of participants’ privacy, but are available from the corresponding author upon reasonable request and with approval from the institutional ethics committee. Requests to access the datasets should be directed to youngmola@foxmail.com.
